# Polygenic risk score-based phenome-wide association study of head and neck cancer across two large biobanks

**DOI:** 10.1186/s12916-024-03305-2

**Published:** 2024-03-14

**Authors:** Young Chan Lee, Sang-Hyuk Jung, Manu Shivakumar, Soojin Cha, Woong-Yang Park, Hong-Hee Won, Young-Gyu Eun, Penn Medicine Biobank, Dokyoon Kim

**Affiliations:** 1 Department of Biostatistics, Epidemiology and Informatics, Perelman School of Medicine, University of Pennsylvania, Philadelphia, PA USA; 2 Department of Otolaryngology-Head and Neck Surgery, School of Medicine, Kyung Hee University, Seoul, Republic of Korea; 3 Hanyang University Institute for Rheumatology Research, Seoul, Republic of Korea; 4 Samsung Genome Institute, Samsung Medical Center, Sungkyunkwan University School of Medicine, Seoul, Republic of Korea; 5 Samsung Medical Center, Samsung Advanced Institute for Health Sciences and Technology, Sungkyunkwan University, Seoul, Republic of Korea; 6 Institute for Translational Medicine and Therapeutics, Perelman School of Medicine, University of Pennsylvania, Philadelphia, PA USA; 7 Institute for Biomedical Informatics, University of Pennsylvania, Philadelphia, PA USA

**Keywords:** Head and neck cancer, Polygenic risk score, Phenome-wide association study, Smoking, Alcohol consumption, Human papillomavirus

## Abstract

**Background:**

Numerous observational studies have highlighted associations of genetic predisposition of head and neck squamous cell carcinoma (HNSCC) with diverse risk factors, but these findings are constrained by design limitations of observational studies. In this study, we utilized a phenome-wide association study (PheWAS) approach, incorporating a polygenic risk score (PRS) derived from a wide array of genomic variants, to systematically investigate phenotypes associated with genetic predisposition to HNSCC. Furthermore, we validated our findings across heterogeneous cohorts, enhancing the robustness and generalizability of our results.

**Methods:**

We derived PRSs for HNSCC and its subgroups, oropharyngeal cancer and oral cancer, using large-scale genome-wide association study summary statistics from the Genetic Associations and Mechanisms in Oncology Network. We conducted a comprehensive investigation, leveraging genotyping data and electronic health records from 308,492 individuals in the UK Biobank and 38,401 individuals in the Penn Medicine Biobank (PMBB), and subsequently performed PheWAS to elucidate the associations between PRS and a wide spectrum of phenotypes.

**Results:**

We revealed the HNSCC PRS showed significant association with phenotypes related to tobacco use disorder (OR, 1.06; 95% CI, 1.05–1.08; *P* = 3.50 × 10^−15^), alcoholism (OR, 1.06; 95% CI, 1.04–1.09; *P* = 6.14 × 10^-9^), alcohol-related disorders (OR, 1.08; 95% CI, 1.05–1.11; *P* = 1.09 × 10^−8^), emphysema (OR, 1.11; 95% CI, 1.06–1.16; *P* = 5.48 × 10^−6^), chronic airway obstruction (OR, 1.05; 95% CI, 1.03–1.07; *P* = 2.64 × 10^−5^), and cancer of bronchus (OR, 1.08; 95% CI, 1.04–1.13; *P* = 4.68 × 10^−5^). These findings were replicated in the PMBB cohort, and sensitivity analyses, including the exclusion of HNSCC cases and the major histocompatibility complex locus, confirmed the robustness of these associations. Additionally, we identified significant associations between HNSCC PRS and lifestyle factors related to smoking and alcohol consumption.

**Conclusions:**

The study demonstrated the potential of PRS-based PheWAS in revealing associations between genetic risk factors for HNSCC and various phenotypic traits. The findings emphasized the importance of considering genetic susceptibility in understanding HNSCC and highlighted shared genetic bases between HNSCC and other health conditions and lifestyles.

**Supplementary Information:**

The online version contains supplementary material available at 10.1186/s12916-024-03305-2.

## Background

Head and neck squamous cell carcinoma (HNSCC), which includes malignancies mainly affecting the oral cavity and oropharynx, holds the position of being the sixth most common cancer worldwide [1, 2]. Tobacco use, including both direct consumption and exposure to smoke, and moderate alcohol intake are accepted as the primary etiological contributors to the development of HNSCC [3]. Infection with human papillomavirus (HPV) also constitutes a significant causative factor, particularly for oropharyngeal cancer (OPC) [4]. However, considering that a significant portion of the evidence concerning these risk factors originates from observational epidemiological studies, it is crucial to examine the underlying associations between risk factors. Moreover, the observation that HNSCC occurrence is limited to a minority among tobacco users, alcohol consumers, and individuals infected with HPV implies a significant involvement of genetic predisposition in its pathophysiology [5]. To achieve this, a comprehensive investigation into the potential involvement of genetic factors is warranted.

Extensive genome-wide association studies (GWASs) have revealed thousands of common variants to be associated with various types of cancer [6]. Polygenic risk scores (PRSs) aim to achieve a substantial improvement in risk prediction by considering the combined effects of multiple risk alleles. These scores provide a valuable methodology for capturing the collective influence of multiple genetic variants, enabling the identification of individuals who are at increased risk of developing site-specific cancers [7]. While the general predictive ability of PRSs for disease outcomes across diverse populations has demonstrated only modest performance in various cancer types, its effectiveness in cohort risk stratification has been substantiated [8, 9]. Recently, utilization of PRSs has expanded to encompass the screening of a diverse array of clinical phenotypes, collectively referred to as the medical phenome, to explore associations of these phenotypes with secondary traits [10].

As a singular biomarker computationally derived from a diverse spectrum of genetic variants, a PRS has markedly greater power than an individual single nucleotide polymorphism (SNP) and can be leveraged to great effect by phenome-wide association studies (PheWAS). PheWAS provide a valuable framework for the simultaneous investigation of genetic variants and physiological and clinical phenotypes, thereby facilitating the exploration of associations across a broad spectrum of traits. In such investigations of the combined landscape of genomics and phenomics, access to both electronic health records (EHRs) and GWAS data is essential.

To date, no studies have been reported that examine the correlation between genetic predisposition to HNSCC and related phenotypes utilizing a PRS-PheWAS analysis. The objective of our study was to demonstrate the potential utility of a PRS derived from a comprehensive population-based GWAS on HNSCC in the prediction of secondary phenotypes within an independent cohort. We conducted PheWAS to examine the correlation between the HNSCC PRS and the EHR-based phenome and validated our findings across independent diverse cohorts. Furthermore, we analyzed the association between HNSCC PRS and lifestyles related to significant phenotypes.

## Methods

### Study population

The UK Biobank (UKBB) is a large prospective observational cohort study that has recruited > 500,000 adults across 22 centers located throughout the UK. The full protocol of the UKBB study is publicly available, and the study design and measurement methods have been described elsewhere [11]. Participants aged 40–69 years were enrolled between 2006 and 2010 and were followed up for subsequent health events. We included in the main analysis individuals diagnosed with International Classification of Diseases (ICD)-9 or ICD-10 codes or identified from hospital episode statistics. All ICD-9 and ICD-10 diagnosis codes and laboratory measurements up to July 2020 were extracted from the EHRs.

The Penn Medicine Biobank (PMBB) is a large academic medical biobank in which participants are agnostically recruited from the outpatient setting and consented for access to their EHR data and permission to generate genomic and biomarker data [12]. The study flowchart is illustrated in Additional file 1: Fig. S1.

### Definition of HNSCC and subtypes

Cancer cases comprised the following ICD-9 codes: oropharynx (145.3, 146.0, and 146.1); oral cavity (140.0–140.9, 141.0–141.9, 142.0–142.8, 143.0–143.9, 144.0–144.9, 145.0–145.9, and 230.0); and larynx (1610–1619), and the following ICD-10 codes: oropharynx (C01, C02.0, C02.4, C05.1, C05.2, C09.0-C10.9, and C14.0), oral cavity (C00.0–C00.9, C02.0–C02.9, C03.0–C03.9, C04.0–C04.9, C05.0–C06.9, and C148), hypopharynx (C12.9, C13.0–C13.2, C13.8, and C13.9), and larynx (C32.0–C32.3, C32.8, and C32.9). The detailed definition criteria for HNSCC and its subtypes in each cohort are described in Additional file 1: Method S2.

### Genotype data quality control and imputation

Genotyping and quality control (QC) procedures and imputation followed standard practices and were performed per cohort-genotyping platform pair. We have filtered out related individuals (with second-degree or closer relatives) by KING software in both biobanks [13]. Further details are described in Additional file 1: Method S3 [14–20].

#### UK Biobank

The UKBB samples (version 3; March 2018) were genotyped for > 800,000 SNPs using either the Affymetrix UK BiLEVE Axiom array or the Affymetrix UKBB Axiom array. After QC and imputation, 308,492 European (White-British) individuals were determined eligible for the validation analyses.

#### Penn Medicine Biobank

The PMBB consists of 43,623 samples that have been genotyped with the GSA genotyping array. After QC and imputation, a total of 27,933 individuals considered of European (non-Hispanic White) ancestry and 10,468 individuals considered of African American (non-Hispanic Black) ancestry were determined eligible for the replication analyses.

### Polygenic risk score

The HNSCC, OPC, and oral cavity cancer (OC) PRSs were generated based on the large-scale HNSCC (5974 cases and 4012 controls), OPC (2617 cases and 4012 controls), and OC (2958 cases and 4012 controls) GWAS summary statistics from the Genetic Associations and Mechanisms in Oncology (GAME-ON) Network (dbGAP [OncoArray: Oral and Pharynx Cancer; study accession number: phs001202.v1.p1]) [21].

To generate the PRSs, we used the Bayesian polygenic prediction method PRS-CS [22]. Individual PRSs were computed from beta coefficients as the weighted sum of the risk alleles by applying PLINK version 1.90 with the --score command [23]. Details of the PRS analysis are described in Additional file 1: Method S4.

### Phenome-wide association study

The *PheWAS* R package (version 0·99·5–5) was used to perform PheWAS analyses [24]. In these analyses, the PRS was set as the independent variable, and disease phenotypes as the dependent variables, with age, sex, genotyping array, and the first 10 genetic principal components (PCs) as covariates. Disease diagnosis category phenotypes were obtained by mapping the ICD-9 and ICD-10 diagnosis codes of the UKBB to 1608 hierarchical phenotypes (PheCodes) categorized into 17 disease categories [24, 25]. We removed phenotypic codes with less than 200 cases and those concerning symptoms, injuries, and poisoning; this left 850 phenotypes in 15 disease categories that were included in our analysis. Of these, 838 were eligible for replication analysis in the PMBB.

### Statistical analysis

Demographic and clinical characteristics are presented as mean ± standard deviation (SD) or as number (percentage). Continuous variables were compared by Student’s *t*-test or the Mann–Whitney *U* test as appropriate. Categorical variables were compared by the chi-square test or Fisher’s exact test as appropriate.

We used a multivariate logistic regression model to evaluate the association of the HNSCC, OPC, and OC PRSs with HNSCC, OPC, and OC occurrence. In the PheWAS analysis, we calculated odds ratios (ORs) and 95% confidence intervals (CIs) after adjusting for age, sex, the first 10 PCs of ancestry, and genotyping array type. The ORs of the PRS were used both as quantitative variables reported per one-SD, and categorical variables were defined as follows: low (0–24th percentile), intermediate (25–49th percentile), high (50–74th percentile), and very high (75–99th percentile). For the PRS-PheWAS analyses, we utilized Bonferroni’s correction for multiple hypothesis testing. We determined *P* < 5.88 × 10^−5^ (= 0.05/850, adjusted for the number of phecode-based traits analyzed in the study) as a statistical significance. In addition, we performed sex, age, and smoking status stratified, HNSCC exclusion, and masked major histocompatibility complex (MHC) regions subgroup sensitivity analyses. Subsequently, we conducted trend analyses to identify statistical differences between the PRS risk group and lifestyles (alcohol use and smoking) and HPV (Additional file 1: Method S5).

All statistical tests were two-sided, and *P* < 0.05 was considered statistically significant. All statistical analyses were conducted using the R Statistical Software (version 4.1.0; R Foundation for Statistical Computing, Vienna, Austria) and PLINK version 1.90 [23].

## Results

### Participants

In total, 308,492 participants of European descent from the UKBB were included, after excluding those having no history of in-patient records or a lack of ICD or self-reported information relevant to this study. The mean age of participants was 58.0 years (SD, 7.9 years). The characteristics of participants in each group are presented in Additional file 1: Table S1. In total, 1763 study subjects had a history of HNSCC, 556 (31.7%) of OPC, and 856 (48.8%) of OC. The “others” category (346 [19.5%]) includes hypopharynx cancer, larynx cancer, and other cancers. Significant differences between the controls and HNSCC cases were observed in HPV positivity, smoking status, and alcohol intake frequency.

For the replication set, a total of 38,401 PMBB participants of European (*n* = 27,933) and African American (*n* = 10,468) descent were included (Additional file 1: Table S2). The mean age of participants was 55.9 years (SD, 16.4 years). Among the HNSCC cases, there were 437 (59.8%) diagnosed with OC, 231 (31.6%) with OPC, and 64 (8.8%) with other cancers.

### PRS association with HNSCC and validation in the UKBB and PMBB

We investigated the associations between PRSs and HNSCC and its subtypes in the UKBB. We observed HNSCC PRS to be associated with the occurrence risk of HNSCC (OR, 1.12; 95% CI, 1.06–1.17; *P* < 0.001), OPC (OR, 1.18; 95% CI, 1.08–1.28; *P* < 0.001), and OC (OR, 1.10; 95% CI, 1.02–1.17; *P* = 0.009). OPC PRS was also associated with occurrence risk of HNSCC (OR, 1.10; 95% CI, 1.05–1.16; *P* < 0.001), OPC (OR, 1.20; 95% CI, 1.10–1.31; *P* < 0.001), but not with OC risk. Meanwhile, OC PRS was associated with the occurrence risk of HNSCC (OR, 1.09; 95% CI, 1.04–1.15; *P* < 0.001), OPC (OR, 1.10; 95% CI, 1.01–1.20; *P* = 0.027), and OC (OR, 1.09; 95% CI, 1.02–1.17; *P* = 0.015) (Additional file 1: Table S3). We also confirmed the association between the PRSs of HNSCC and its subtypes with the risk of occurrence in subgroups based on age, sex, and smoking status (Additional file 1: Table S4). These associations were replicated in the PMBB cohort: the PRSs for HNSCC and OPC showed significant association with HNSCC and its subtypes, while that for OC exhibited weaker association (Additional file 1: Table S5). We estimated the proportion of variance explained by the PRSs for HNSCC, OPC, and OC in both cohorts (Additional file 1: Table S3 and S5).

In order to investigate the impact of unbalanced case-to-control ratios between the two cohorts, we expanded our analysis of the PRS at different ratios across data from both biobanks (Additional file 1: Table S6). In addition, we performed ancestry-specific analyses in the PMBB (Additional file 1: Table S7). We found that the inherent differences in the characteristics of the target cohorts could potentially impact the performance of the PRS analysis, including the proportion of variance explained, regardless of identical proportions.

### PRS-PheWAS

**Fig. 1:**
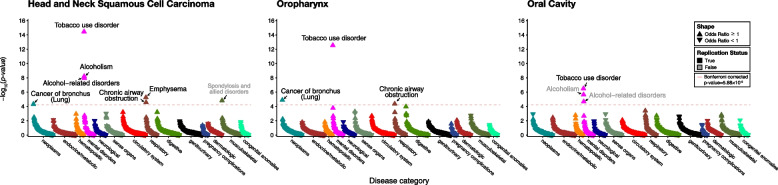
PheWAS Manhattan plot of HNSCC and subtypes genetic risk score in UK Biobank. Abbreviations: PheWAS, phenome-wide association study; HNSCC, head and neck squamous cell carcinoma

We tested the association between HNSCC PRS and phenotypes constructed in the UKBB (Fig. 1). In HNSCC PRS, the strongest association was observed for “Tobacco use disorder” (OR, 1.06; 95% CI, 1.05–1.08; *P* = 3.50 × 10^−15^). The HNSCC PRS was also associated with “Alcoholism” (OR, 1.06; 95% CI, 1.05–1.09; *P* = 6.14 × 10^−9^), “Alcohol-related disorders” (OR, 1.08; 95% CI, 1.04–1.09; *P* = 1.09 × 10^−8^), “Emphysema” (OR, 1.11; 95% CI, 1.06–1.16; *P* = 5.48 × 10^−6^), “Chronic airway obstruction” (OR, 1.05; 95% CI, 1.03–1.07; *P* = 2.64 × 10^−5^), “Cancer of bronchus; lung” (OR, 1.08; 95% CI, 1.04–1.13; *P* = 4.68 × 10^−5^), and “Spondylosis and allied disorders” (OR, 1.05; 95% CI, 1.03–1.07; *P *= 1.46 × 10^-5^) (Table 1 and Additional file 2: Table S8).

**Table 1:** Significant associations of HNSCC PRS with PheWAS in the UK Biobank that were also replicated in the Penn Medicine Biobank

**Phenotype description**	**UK Biobank**^**a**^			**Penn Medicine Biobank**^**b**^ (replication cohort)	
	**No. of cases (prevalence, %)**	**OR per SD increase (95% CI)**	***P*-value**	**OR per SD increase (95% CI)**	***P*-value**
**HNSCC PRS**					
Tobacco use disorder	20,599 (6.7%)	1.06 (1.05–1.08)	3.50 × 10^−15^	1.04 (1.02–1.07)	1.05 × 10^−3^
Alcoholism	9636 (3.1%)	1.06 (1.04–1.09)	6.14 × 10^−9^	1.11 (1.01–1.22)	2.29 × 10^−2^
Alcohol-related disorders	6015 (1.9%)	1.08 (1.05–1.11)	1.09 × 10^−8^	1.11 (1.02–1.22)	2.22 × 10^−2^
Emphysema	2096 (0.7%)	1.11 (1.06–1.16)	5.48 × 10^−6^	1.10 (1.03–1.16)	2.44 × 10^−3^
Chronic airway obstruction	9151 (3.0%)	1.05 (1.03–1.07)	2.64 × 10^−5^	1.08 (1.04–1.11)	2.16 × 10^−5^
Cancer of bronchus; lung	2781 (0.9%)	1.08 (1.04–1.13)	4.68 × 10^−5^	1.12 (1.05–1.19)	4.44 × 10^−4^
**OPC PRS**					
Tobacco use disorder	20,599 (6.7%)	1.06 (1.04–1.07)	2.85 × 10^−13^	1.04 (1.02–1.07)	1.43 × 10^−3^
Cancer of bronchus; lung	2781 (0.9%)	1.09 (1.05–1.13)	1.27 × 10^−5^	1.07 (1.01–1.14)	2.05 × 10^−2^
Chronic airway obstruction	9151 (3.0%)	1.05 (1.02–1.07)	4.32 × 10^−5^	1.04 (1.01–1.08)	1.20 × 10^−2^
**OC PRS**					
Tobacco use disorder	20,599 (6.7%)	1.04 (1.02–1.05)	3.20 × 10^−7^	1.04 (1.02–1.07)	1.48 × 10^−3^

*Abbreviations: HNSCC*, head and neck squamous cell carcinoma; *OC*, oral cavity cancer; *OPC*, oropharynx; *PRS*, polygenic risk score; *SD*, standard deviation; *OR*, odds ratio; *CI*, confidence interval; *PC*, principal component

^a^Multivariable regression analysis was adjusted by age, sex, genotype array, and PC 1 to 10

^b^Multivariable regression analysis was adjusted by age, sex, ethnicity, and PC 1 to 10

In the subtype PRS analysis for OPC PRS, the phenotype most strongly associated was “Tobacco use disorder,” followed by “Cancer of bronchus; lung” and “Chronic airway obstruction” (Table 1 and Additional file 2: Table S9). Meanwhile, for the OC PRS, significant associations were observed with “Tobacco use disorder,” “Alcoholism,” and “Alcohol-related disorders” (Table 1 and Additional file 2: Table S10). When stratified by HNSCC PRS percentile, we confirmed the prevalence of each phenotype to be increased with higher PRS percentiles (Additional file 1: Fig. S2).

### PRS-PheWAS validation in the PMBB

To establish the correlation of PRSs with the identified phenotype traits, we replicated the association analyses within the corresponding phenotype of the PMBB dataset. Upon examination of the PMBB phenome, the majority of previously observed associations were validated; the exceptions were the traits “Spondylosis and allied disorders” with the HNSCC PRS and “Alcoholism” and “Alcohol-related disorders” with the OC PRS, which did not exhibit significant associations (Table 1 and Additional file 2: Tables S8-10).

### Sensitivity analysis

#### Exclusion PheWAS

To investigate whether the observed associations of the HNSCC PRS with phenotypes were solely attributable to the inclusion of HNSCC cases, we conducted a PheWAS after excluding HNSCC cases from the UKBB. We still found consistent associations between HNSCC PRS and the phenotypes after removing 1753 HNSCC case subjects compared to the full analysis. Specifically, “Tobacco use disorder,” “Alcoholism,” “Alcohol-related disorders,” “Emphysema,” “Chronic airway obstruction,” and “Cancer of bronchus; lung” remained significantly associated with HNSCC PRS in the UKBB (Table 2).

**Table 2:** Sensitivity analysis results of HNSCC PRS with significant associations in the UK Biobank

**Phenotype description**	**Exclusion analysis**^**1**^		**Exclusion analysis**^**2**^		**Exclusion analysis**^**3**^	
	**OR per SD increase (95% CI)**	***P*-value**	**OR per SD increase (95% CI)**	***P*-value**	**OR per SD increase (95% CI)**	***P*-value**
**HNCSS PRS**						
Tobacco use disorder	1.06 (1.04–1.07)	6.83 × 10^−14^	1.06 (1.05–1.08)	3.41 × 10^−15^	1.06 (1.04–1.07)	6.84 × 10^−14^
Alcoholism	1.06 (1.04–1.08)	5.38 × 10^−8^	1.06 (1.04–1.09)	6.09 × 10^−9^	1.06 (1.04–1.08)	5.28 × 10^−8^
Alcohol-related disorders	1.08 (1.05–1.11)	6.98 × 10^−8^	1.08 (1.05–1.11)	1.04 × 10^−8^	1.08 (1.05–1.11)	6.57 × 10^−8^
Emphysema	1.11 (1.06–1.16)	9.15 × 10^−6^	1.11 (1.06–1.16)	4.92 × 10^−6^	1.11 (1.06–1.16)	8.41 × 10^−6^
Chronic airway obstruction	1.04 (1.02–1.07)	1.20 × 10^−4^	1.05 (1.02–1.07)	2.70 × 10^−5^	1.04 (1.02–1.07)	1.12 × 10^−4^
Cancer of bronchus; lung	1.08 (1.04–1.13)	6.41 × 10^−5^	1.08 (1.04–1.13)	5.01 × 10^−5^	1.08 (1.04–1.13)	6.90 × 10^−5^
**OPC PRS**						
Tobacco use disorder	1.05 (1.04–1.07)	5.51 × 10^−12^	1.05 (1.04–1.07)	2.72 × 10^−12^	1.05 (1.04–1.07)	2.44 × 10^−11^
Cancer of bronchus; lung	1.09 (1.05–1.13)	2.30 × 10^−5^	1.09 (1.05–1.13)	1.62 × 10^−5^	1.09 (1.05–1.13)	2.49 × 10^−5^
Chronic airway obstruction	1.04 (1.02–1.07)	1.62 × 10^−4^	1.04 (1.02–1.07)	1.06 × 10^−4^	1.04 (1.02–1.06)	2.99 × 10^−4^
**OC PRS**						
Tobacco use disorder	1.04 (1.02–1.05)	1.36 × 10^−6^	1.04 (1.02–1.05)	2.42 × 10^−7^	1.04 (1.02–1.05)	9.64 × 10^−7^

*Abbreviations: HNSCC*, head and neck squamous cell carcinoma; *OC*, oral cavity cancer; *OPC*, oropharynx cancer; *PRS*, polygenic risk score; *MHC*, major histocompatibility complex; *SD*, standard deviation; *OR*, odds ratio; *CI*, confidence interval; *PC*, principal component

All analyses were adjusted by age, sex, genotype array, and PC 1 to 10

^1^Exclusion analysis in which HNSCC cases were excluded from PheWAS

^2^Exclusion analysis in which the MHC region was excluded from PRS generation

^3^Exclusion analysis in which the MHC region was excluded from PRS generation and HNSCC cases were excluded from PheWAS

#### MHC region exclusion analysis

We also generated a HNSCC PRS excluding MHC locus. We observed this score to exhibit a persistent significant association with all phenotypes even after excluding the entire MHC region. Moreover, these significant correlations remained in a second sensitivity analysis that further excluded HNSCC cases as well as the MHC region (Table 2).

#### Sex, age, and smoking status-stratified analyses

In sex-stratified analysis, all phenotypes remained significant. Overall, there was no significant sex interaction (Table 3). There was no significant association between “Cancer of bronchus; lung” and HNSCC PRS in the younger (age ≤ 60 years) group, while all phenotypes showed significant associations in the elderly group (age > 60 years). In addition, “Alcoholism” and “Emphysema” were only significant in the never-smoker group, while all phenotypes showed significant associations in the ever-smoker group (Table 4).

**Table 3:** Sex-stratified results of HNSCC PRS with significant associations in the UK Biobank

**Phenotype description**		**Male (***n* **= 140,232)**		**Female (***n* **= 168,260)**		**Sex-interaction**
	**No. of cases (%)**	**OR per SD increase (95% CI)**	***P*-value**	**OR per SD increase (95% CI)**	***P*-value**	***P*-value for interaction**
**HNSCC PRS**						
Tobacco use disorder	20,599 (6.7%)	1.06 (1.04–1.08)	3.06 × 10^−8^	1.07 (1.04–1.09)	1.26 × 10^−8^	.207
Alcoholism	9636 (3.1%)	1.06 (1.03–1.09)	1.44 × 10^−5^	1.07 (1.03–1.11)	9.08 × 10^−5^	.886
Alcohol-related disorders	6015 (1.9%)	1.08 (1.05–1.12)	5.95 × 10^−7^	1.07 (1.02–1.13)	4.35 × 10^−3^	.830
Emphysema	2096 (0.7%)	1.10 (1.04–1.16)	1.50 × 10^−3^	1.13 (1.05–1.21)	9.79 × 10^−4^	.470
Chronic airway obstruction	9151 (3.0%)	1.04 (1.02–1.08)	2.95 × 10^−3^	1.05 (1.02–1.09)	2.94 × 10^−3^	.591
Cancer of bronchus; lung	2781 (0.9%)	1.10 (1.05–1.16)	2.74 × 10^−4^	1.06 (1.00–1.12)	4.04 × 10^−2^	.365

Analyses were adjusted by age, genotype array, and PC 1 to 10

*Abbreviations: HNSCC* head and neck squamous cell carcinoma, *OC* oral cavity cancer, *OPC* oropharynx cancer, *PRS* polygenic risk score, *SD* standard deviation, *OR* odds ratio, *CI* confidence interval, *PC* principal component

**Table 4:** Subgroup-stratified results of HNSCC PRS with significant associations in the UK Biobank

**Phenotype description**	**Younger (age ≤ 60 years)**		**Elderly (age > 60 years)**		**Never-smoker**		**Ever-smoker**	
	**(*n* = 166,624)**		**(*n* = 141,868)**		**(*n* = 119,038)**		**(*n* = 190,562)**	
	**OR per SD increase (95% CI)**	***P*-value**	**OR per SD increase (95% CI)**	***P*-value**	**OR per SD increase (95% CI)**	***P*-value**	**OR per SD increase (95% CI)**	***P*-value**
**HNSCC PRS**								
Tobacco use disorder	1.08 (1.06–1.10)	1.54 × 10^−13^	1.04 (1.02–1.06)	3.77 × 10^−4^	1.02 (0.94–1.10)	.631	1.06 (1.04–1.07)	3.11 × 10^−12^
Alcoholism	1.07 (1.04–1.10)	1.16 × 10^−5^	1.06 (1.03–1.09)	1.38 × 10^−4^	1.05 (1.00–1.09)	3.09 × 10^−2^	1.07 (1.04–1.10)	8.80 × 10^−8^
Alcohol-related disorders	1.09 (1.05–1.13)	6.24 × 10^−7^	1.07 (1.02–1.11)	3.44 × 10^−3^	1.04 (0.98–1.11)	.149	1.08 (1.05–1.12)	8.20 × 10^−8^
Emphysema	1.13 (1.04–1.22)	3.22 × 10^−3^	1.10 (1.04–1.16)	4.34 × 10^−4^	1.26 (1.07–1.48)	4.97 × 10^−3^	1.09 (1.04–1.14)	3.70 × 10^−4^
Chronic airway obstruction	1.06 (1.02–1.10)	6.13 × 10^−3^	1.04 (1.02–1.07)	1.25 × 10^−3^	0.99 (0.93–1.06)	.864	1.05 (1.02–1.07)	6.61 × 10^−5^
Cancer of bronchus; lung	1.06 (0.99–1.14)	7.86 × 10^−2^	1.09 (1.04–1.14)	1.90 × 10^−4^	1.05 (1.01–1.09)	.355	1.08 (1.04–1.13)	1.25 × 10^−4^

All analyses were adjusted by age, sex, genotype array, and PC 1 to 10

*Abbreviations: HNSCC* head and neck squamous cell carcinoma, *PRS* polygenic risk score, *SD* standard deviation, *OR* odds ratio, *CI* confidence interval, *PC* principal component

### Association between HNSCC PRS and smoking, alcohol consumption, and HPV seropositivity

As we observed HNSCC PRS to have associations with the phenotypes of alcoholism and smoking, which were generated based on ICD codes, we proceeded to explore its connections with lifestyle factors related to actual alcohol consumption and smoking. Having a very high PRS was significantly associated with current smoking status (*P* < 0.001), previously smoked a high number of cigarettes daily (*P* < 0.001), high pack years of smoking (*P* < 0.001), past tobacco smoking (*P* < 0.001), maternal smoking around birth (*P* < 0.001), stopped smoking at a high age (*P* < 0.001), and a high number of unsuccessful stop-smoking attempts (*P* = 0.006) (Table 5). We also observed significant associations of HNSCC PRS with alcohol drinker status (*P* < 0.001), frequency (*P* = 0.045), amount (*P* < 0.001), alcohol usually taken with meals (*P* < 0.001), and a history of past alcohol consumption (*P* < 0.001) (Table 6). However, no significant association was found between HNSCC PRS and seropositivity for HPV type-16 (Table 7).

**Table 5:** Smoking-related characteristics according to the genetic risk group of HNSCC

	**Low** **genetic risk** **group** **(0th–24th)**	**Intermediate genetic risk group** **(25th–49th)**	**High** **genetic risk group** **(50th–74th)**	**Very high** **genetic risk group** **(75th–99th)**	***P*-value**
	(*n* = 76,502)	(*n* = 77,180)	(*n* = 77,142)	(*n* = 77,668)	
**Status**					
Smoking status (UKBB field: 20116), No. (%)					< .001
Never	41,282 (54.2%)	40,837 (53.1%)	40,711 (53.0%)	39,893 (51.6%)	
Previous	27,655 (36.3%)	28,229 (36.7%)	28,071 (36.5%)	28,367 (36.7%)	
Current	7270 (9.5%)	7834 (10.2%)	8099 (10.5%)	9101 (11.8%)	
Current tobacco smoking (UKBB field: 1239), No. (%)					< .001
No	69,176 (90.5%)	69,291 (89.8%)	68,988 (89.5%)	68,527 (88.3%)	
Only occasionally	2001 (2.6%)	1997 (2.6%)	2012 (2.6%)	2096 (2.7%)	
Yes	5269 (6.9%)	5837 (7.6%)	6087 (7.9%)	7005 (9.0%)	
**Amount**					
Number of cigarettes previously smoked daily (UKBB field: 2887), mean ± SD	19.0 ± 10.5	19.5 ± 10.7	19.4 ± 10.6	19.7 ± 10.8	< .001
Pack years of smoking (UKBB field: 20161), mean ± SD	23.6 ± 18.9	24.1 ± 19.2	24.5 ± 19.5	25.3 ± 19.7	< .001
**History**					
Past tobacco smoking (UKBB field: 1249), No. (%)					< .001
Smoked on most or all days	18,951 (26.6%)	19,780 (27.7%)	19,716 (27.8%)	20,420 (28.9%)	
Smoked occasionally	10,381 (14.6%)	10,178 (14.3%)	10,112 (14.2%)	9833 (13.9%)	
Just tried once or twice	11,203 (15.7%)	10,801 (15.1%)	10,621 (15.0%)	10,066 (14.3%)	
I have never smoked	30,603 (43.0%)	30,542 (42.8%)	30,584 (43.1%)	30,284 (42.9%)	
Maternal smoking around birth (UKBB field: 1787), No. (%)					< .001
No	46,341 (70.3%)	45,699 (68.8%)	44,992 (67.9%)	43,882 (66.0%)	
Yes	19,532 (29.7%)	20,716 (31.2%)	21,318 (32.1%)	22,587 (34.0%)	
Age stopped smoking (UKBB field: 2897), mean ± SD	40.3 ± 11.9	40.3 ± 11.9	40.5 ± 11.8	40.7 ± 11.9	< .001
Number of unsuccessful stop-smoking attempts (UKBB field: 2926), mean ± SD	2.9 ± 7.0	3.0 ± 6.8	3.0 ± 7.9	3.1 ± 7.5	.006

*Abbreviations: HNSCC* head and neck squamous cell carcinoma, *UKBB* UK Biobank

**Table 6:** Alcohol-related characteristics according to the genetic risk group of HNSCC

	**Low** **genetic risk group** **(0th–24th)**	**Intermediate genetic risk group** **(25th–49th)**	**High** **genetic risk group** **(50th–74th)**	**Very high genetic risk group (75th–99th)**	***P*-value**
	(*n* = 76,502)	(*n* = 77,180)	(*n* = 77,142)	(*n* = 77,668)	
**Status**					
Alcohol drinker status (UKBB field: 20117), No. (%)					.001
Never	2501 (3.3%)	2455 (3.2%)	2525 (3.3%)	2468 (3.2%)	
Previous	2677 (3.5%)	2770 (3.6%)	2888 (3.7%)	3023 (3.9%)	
Current	71,245 (93.2%)	71,879 (93.2%)	71,652 (93.0%)	72,077 (92.9%)	
Alcohol intake frequency (UKBB field: 1558), No. (%)					.045
Daily or almost daily	14,927 (19.5%)	15,235 (19.7%)	15,082 (19.6%)	15,158 (19.5%)	
Three or four times a week	17,599 (23.0%)	17,756 (23.0%)	17,756 (23.0%)	18,049 (23.2%)	
Once or twice a week	20,490 (26.8%)	20,416 (26.5%)	20,451 (26.5%)	20,856 (26.9%)	
One to three times a month	8867 (11.6%)	9087 (11.8%)	8949 (11.6%)	8775 (11.3%)	
Special occasions only	8989 (11.8%)	8997 (11.7%)	9007 (11.7%)	8894 (11.5%)	
Never	5617 (7.3%)	5677 (7.4%)	5886 (7.6%)	5927 (7.6%)	
**Amount**					
Amount of alcohol drunk on a typical drinking day (UKBB field: 20403), No. (%)					< .001
1 or 2	12,035 (53.5%)	11,304 (51.9%)	11,374 (52.3%)	10,423 (49.6%)	
3 or 4	6031 (26.8%)	5958 (27.4%)	5797 (26.6%)	5823 (27.7%)	
5 or 6	2530 (11.2%)	2544 (11.7%)	2554 (11.7%)	2596 (12.4%)	
7, 8 or 9	1322 (5.9%)	1319 (6.1%)	1387 (6.4%)	1542 (7.3%)	
10 or more	572 (2.5%)	650 (3.0%)	645 (3.0%)	622 (3.0%)	
Frequency of consuming six or more units of alcohol (UKBB field: 20416), No. (%)					< .001
Never	11,933 (52.9%)	11,265 (51.6%)	11,200 (51.3%)	10,463 (49.7%)	
Less than monthly	5409 (24.0%)	5155 (23.6%)	5286 (24.2%)	5014 (23.8%)	
Monthly	1854 (8.2%)	1870 (8.6%)	1770 (8.1%)	1876 (8.9%)	
Weekly	2647 (11.7%)	2779 (12.7%)	2772 (12.7%)	2895 (13.8%)	
Daily or almost daily	702 (3.1%)	753 (3.5%)	790 (3.6%)	800 (3.8%)	
**Type**					
Alcohol usually taken with meals (UKBB field: 1618), No. (%)					< .001
No	12,880 (30.9%)	13,435 (32.5%)	13,721 (33.4%)	14,685 (35.8%)	
Yes	28,817 (69.1%)	27,866 (67.5%)	27,384 (66.6%)	26,379 (64.2%)	
Other non-alcoholic drinks (UKBB field: 100510), No. (%)					.330
No	26,030 (78.1%)	25,290 (78.3%)	24,752 (78.0%)	24,012 (78.6%)	
Yes	7284 (21.9%)	7003 (21.7%)	6973 (22.0%)	6540 (21.4%)	
**History**					
Alcohol intake versus 10 years previously (UKBB field: 1628), No. (%)					< .001
More nowadays	10,268 (14.5%)	10,477 (14.7%)	10,474 (14.7%)	11,148 (15.6%)	
About the same	26,488 (37.4%)	26,201 (36.6%)	25,685 (36.1%)	24,997 (34.9%)	
Less nowadays	34,083 (48.1%)	34,822 (48.7%)	35,084 (49.2%)	35,518 (49.6%)	
More nowadays	10,268 (14.5%)	10,477 (14.7%)	10,474 (14.7%)	11,148 (15.6%)	
Ever physically dependent on alcohol (UKBB field: 20404), No. (%)					.006
No	369 (74.7%)	404 (73.1%)	377 (69.4%)	373 (65.8%)	
Yes	125 (25.3%)	149 (26.9%)	166 (30.6%)	194 (34.2%)	
Ever had known a person concerned about, or recommended reduction of, alcohol consumption (UKBB field: 20405), No. (%)					< .001
No	22,649 (91.9%)	21,921 (91.3%)	21,753 (91.2%)	20,823 (90.6%)	
Yes, but not in the last year	1046 (4.2%)	1088 (4.5%)	1118 (4.7%)	1184 (5.1%)	
Yes, during the last year	941 (3.8%)	992 (4.1%)	987 (4.1%)	989 (4.3%)	

*Abbreviations: HNSCC* head and neck squamous cell carcinoma, *UKBB* UK Biobank

**Table 7:** HPV characteristics according to the genetic risk group of HNSCC

	**Low genetic risk group (0th–24th)**	**Intermediate genetic risk group (25th–49th)**	**High genetic risk group (50th–74th)**	**Very high genetic risk group (75th–99th)**	***P*-value**
	**(*n* = 76,502)**	**(*n* = 77,180)**	**(*n* = 77,142)**	**(*n* = 77,668)**	
**Status**					
HPV type-16 (UKBB field: 23075), No. (%)					.768
Positive	69 (4.8%)	76 (5.1%)	66 (4.4%)	65 (4.4%)	
Negative	1366 (95.2%)	1424 (94.9%)	1445 (95.6%)	1421 (95.6%)	

*Abbreviations: HNSCC* head and neck squamous cell carcinoma, *HPV* human papillomavirus, *UKBB* UK Biobank

## Discussion

The aim of this study was to explore phenotypes connected to the genetic predisposition for HNSCC within the UKBB cohort, for which we utilized a PheWAS. These findings were validated in a replication set involving 38,401 participants from the PMBB.

The HNSCC PRS constructed here, including subtypes such as OC and OPC, incorporated the most extensive assemblage of SNPs discovered in the recent GWAS for HNSCC conducted by the GAME-ON Network [21]. The resultant PRS was robustly validated in both the UKBB (European) and the PMBB (European and African American) cohorts, despite the population diversity present within the PMBB. One previous study derived PRSs for 16 cancer types, including a HNSCC PRS derived from the 14 SNPs in prior HNSCC GWASs; this PRS demonstrated the most minimal effect size with an OR of 1.08 [26]. Another PRS based on summary data from the FinnGen HNSCC GWAS showed a nonsignificant association with the risk of HNSCC [27]. Our validated and replicated results and higher OR of 1.17 (95% CI, 1.07–1.26) indicate improved performance of the HNSCC PRS for capturing high-risk individuals. The two datasets used to evaluate and validate the performance of the HNSCC PRS are cohorts with distinct characteristics and diverse ancestry. The UKBB is a prospective national cohort study based on healthy participants, whereas the PMBB is an academic research cohort derived from a regional university hospital with diverse ancestry. Therefore, although these datasets differ in their case–control ratios, when analyzed with an alternative ratio, they demonstrated differences in the proportion of variance explained. This suggests that the distinct characteristics of the different cohorts and ancestry influence the results of the performance analysis of the PRS.

The overall low effect of the HNSCC PRS can be attributed to several factors. Firstly, the etiology of HNSCC is multifaceted, involving a complex interplay of genetic, environmental, and lifestyle factors. While PRSs are designed to capture the cumulative effect of multiple genetic variants, they might not fully account for the intricate interactions between genetic variations and the diverse array of risk factors specific to PRS. Additionally, the genetic architecture of HNSCC might not be as strongly influenced by common variants as some other diseases [28]. This could result in the PRS having lower predictive power, as it relies heavily on the contributions of common variants. Furthermore, the HNSCC patient group is heterogeneous, which poses a distinct challenge. Cancers at different subsites within the head and neck region (e.g., oral cavity, pharynx, and larynx) may have distinct genetic underpinnings and risk factors, making it harder for a general PRS to accurately predict risk across all subtypes. On the other hand, PRSs can serve as a valuable tool for conducting PheWAS to unveil secondary trait associations facilitated by the presence of shared genetic risk factors. These secondary associations have the potential to unveil characteristics within EHRs that manifest prior to cancer diagnosis, and hence could emerge as meaningful predictors for cancer outcomes [27]. Fritsche et al. conducted a comprehensive PheWAS using PRSs encompassing 35 prevalent cancer traits; however, their analysis did not yield any substantial phenotypic associations for oral cancer and laryngeal cancer, the examined types that correspond to HNSCC [27]. Our study explored the associations between HNSCC PRS and various phenotypes constructed from the UKBB cohort. Notably, we observed strong associations of the PRS with certain phenotypes; for instance, “Tobacco use disorder” showed a particularly robust association, indicating the importance of smoking as a risk factor for HNSCC. This association was also detected when using both OPC and OC PRSs. Additional associations with “Alcoholism,” “Alcohol-related disorders,” and other health conditions suggest a complex interplay of lifestyle and genetic factors in HNSCC risk and particularly imply that HNSCC and disorders related to alcohol and smoking share a genetic basis. A case–control study also reported polymorphism in glutathione S-transferase genes and interaction with environmental factors such as smoking and alcohol on susceptibility to HNSCC [29].

In a previous Mendelian randomization (MR) analysis, researchers observed a PRS representing genetic susceptibility to smoking initiation to be non-significantly associated with elevated risk of HNSCC [30]. Another study conducted univariable and multivariable MR analyses utilizing summary-level genetic data from the GWAS and Sequencing Consortium of Alcohol and Nicotine Use, the UKBB, and the GAME-ON Network, which revealed independent causal impacts of both smoking and alcohol on the risk of oral and OPC [31].

Smoking is notably correlated with the prevalence of HNSCC [32], and this association is particularly evident in cases involving tumors originating from the oral cavity, nasopharynx, oropharynx, hypopharynx, and larynx [33]. Some genetic variations might contribute to both increased HNSCC risk and a higher susceptibility to smoking addiction [34]. In particular, certain genes related to nicotine metabolism, neurotransmitter pathways, and cellular processes can influence both smoking behavior and cancer susceptibility [35]. A recent study also found that genetic variants in metabolic genes linked to polycyclic aromatic hydrocarbons and tobacco-specific nitrosamines exhibit associations with susceptibility to HNSCC and its subtypes [36]. Moreover, findings from prior PheWAS have revealed significant correlations between these genes and the risks of diverse cancers, along with smoking behavior. Meanwhile, when it comes to alcohol consumption, observational evidence regarding connections with different types of cancers presents varying conclusions [37]. The interaction of genetic polymorphisms related to alcohol metabolism with alcohol drinking has been noted to affect the risk of HNSCC [38]. In particular, Chien et al. showed SNPs in genes encoding alcohol-metabolizing enzymes (*ADH1B*, *ADH1C*, and *ALDH2*) to be associated with patients’ susceptibility to developing multiple primary tumors, especially in the hypopharynx and esophagus, which are challenging in patients with HNC [39]. Our findings add to these reports by unveiling the association of HNSCC PRS with smoking and alcohol-related disorder.

Graff et al. previously explored the presence of PRS-specific pleiotropy across 16 types of cancer using individuals of European ancestry from the Genetic Epidemiology Research on Adult Health and Aging cohort and the UKBB [26]. In their findings, lung cancer PRS was positively associated with oral/pharyngeal cancer, but oral/pharyngeal cancer PRS was inversely associated with lung cancer. This inconsistency could be attributed to two specific variants (rs467095 and rs10462706) among the 14 associated with oral/pharyngeal sites, which were inversely correlated with lung cancer risk. Meanwhile, the HNSCC PRS in this study, which was based on hundreds of thousands of variants through the PRS-CS approach, showed significant positive pleiotropy with cancer of the bronchus, chronic airway obstruction, and emphysema. A recent study showed that SNP (rs3017895 located in the *FAM13A*) may contribute to OC, which had a strong association with chronic obstructive lung disease including emphysema in GWAS [40].

In this study, we conducted several sensitivity analyses to assess the robustness of our findings, including sex, age, and smoking status stratified assessments, exclusion analyses, and exclusion of the MHC region. That last analysis was conducted due to several MHC risk variants, particularly the class II HLA genes (e.g., *HLA-DPB1*), having a known substantial impact on genetic predisposition to HNSCC [21, 41]. As a result, the identified associations were consistent across sensitivity analyses, providing further confidence in the study’s results. Moreover, the analyses excluding MHC variants consistently showed similar effect sizes, indicating a restricted role of such variants in HNSCC.

Cancer susceptibility is multifaceted, encompassing not only genetic risk factors but also various lifestyle, anthropometric, hormonal, reproductive, and imaging factors [42]. In the context of our study, the prediction of HNSCC based solely on genetic factors proves challenging, given the multifactorial nature of cancer, the involvement of numerous genes, the impact of environmental factors, and the incomplete elucidation of the intricate interplay between genetics and non-genetic risk factors. Our results, derived from the establishment of HNSCC PRS within a relatively extensive cohort, reveal an association with the disease across two cohorts. However, the predictive efficacy was relatively low. Notably, through PRS-PheWAS, our investigation confirmed a significant correlation between HNSCC and disease entities related to alcohol and smoking, which are well-known modifiable risk factors for HNSCC. We analyzed the association between genetic risk and the major risk factors for HNSCC, such as alcohol and tobacco-related lifestyle habits and HPV infection. We found high PRS risk to be significantly associated with various smoking-related characteristics, including current smoking status, pack years of smoking, and age at smoking cessation. This reinforces the well-established link between smoking and HNSCC risk. Similarly, the study identified significant associations with alcohol-related factors, such as alcohol drink status and past alcohol consumption. Taken together, these findings emphasize the roles of smoking and alcohol consumption as risk factors for HNSCC. However, no significant association was found between HNSCC PRS and seropositivity for HPV type-16. Considering the limited sample size for HPV seropositive and seronegative cases in the UKBB, it becomes challenging to draw definitive conclusions regarding the correlation between HNSCC PRS and HPV seropositivity. Our investigation establishes significant associations between genetic and modifiable risk factors for HNSCC within a population-based cohort, distinguished by a comprehensive dataset encompassing diverse phenotypes and cancer risk factors. By identifying these associated secondary phenotypes, we could understand the genetic factors in HNSCC better and improve the prediction ability for HNSCC by considering interactions with various non-genetic traits in the future [43, 44].

### Limitations

This study has several limitations. Firstly, despite conducting numerous sensitivity analyses, the possibility of pleiotropic effects resulting from multiple genetic instruments cannot be eliminated unless all the biological impacts of each and every SNP are comprehensively understood. Secondly, HNSCC is a markedly heterogeneous malignancy, encompassing molecular subtypes that exhibit contrasting behaviors [45]. Adopting a broader phenotype definition would permit larger sample sizes, but it could also lead to the inclusion of genetically diverse phenotypes, contributing to increased disease heterogeneity and a subsequent reduction in predictive capability [46]. Conversely, refining the phenotype might enhance homogeneity, but it could constrain sample size, with consequent loss of statistical power.

## Conclusions

In conclusion, this study provides valuable insight into the genetic risk factors associated with HNSCC and its subtypes. The findings highlight the importance of PRS as a tool for understanding disease risk and suggest a complex interaction between genetic susceptibility and lifestyle factors, particularly smoking and drinking. These findings have the potential to inform strategies for HNSCC prevention and personalized medicine. Further research may be needed to explore the underlying mechanisms linking genetics, lifestyle, and HNSCC risk in more detail.

### Supplementary Information

**Additional file 1:** **Method S1.** Penn Medicine Biobank banner author list and contribution statements. **Method S2.** Detailed definition of HNSCC. **Method S3.** Detailed information on the genotype data quality control and imputation procedures. **Method S4.** Generation of polygenic risk scores. **Method S5.** Number of missing data for each variable in the UK Biobank. **Table S1.** Characteristics of participants in the UK Biobank. **Table S2.** Characteristics of participants in the Penn Medicine Biobank. **Table S3.** Odds ratio for HNSCC and its subtypes associated with genetic risk in the UK Biobank. **Table S4.** Odds ratio for HNSCC and its subtypes associated with genetic risk across subgroups by age, sex, and smoking status in the UK Biobank. **Table S5.** Odds ratio for HNSCC and its subtypes associated with genetic risk in the Penn Medicine Biobank. **Table S6.** Odds ratio for HNSCC associated with genetic risk across different case–control ratios in the UK Biobank and Penn Medicine Biobank. **Table S7.** The ancestry-specific odds ratio for HNSCC associated with genetic risk in the Penn Medicine Biobank. **Figure S1.** Study flowchart. **Figure S2.** Prevalence plot for significant phenotypes in PheWAS according to genetic risk groups.

**Additional file 2:** **Table S8.** Full results of HNSCC PRS-PheWAS in UK Biobank and Penn Medicine Biobank. **Table S9.** Full results of OPC PRS-PheWAS in UK Biobank and Penn Medicine Biobank. **Table S10.** Full results of OC PRS-PheWAS in UK Biobank and Penn Medicine Biobank.

## Data Availability

GAME-ON Network data, including HNSCC and its subtypes genotype and phenotype data, are available for download from the dbGAP upon appropriate request under study accession number phs001202.v1.p1 (OncoArray: Oral and Pharynx Cancer, https://www.ncbi.nlm.nih.gov/projects/gap/cgi-bin/study.cgi?study_id=phs001202.v1.p1). The HNSCC, OPC, and OC PRS models constructed in the current paper are available for download from the GitHub page (https://github.com/dokyoonkimlab/hnc-prs-phewas).

## Abbreviations

CI: Confidence interval
GAME-ON: Genetic Associations and Mechanisms in Oncology
GWAS: Genome-wide association study
HER: Electronic health records
HNSCC: Head and neck squamous cell carcinoma
HPV: Human papillomavirus
ICD: International Classification of Diseases
MHC: Major histocompatibility complex
MR: Mendelian randomization
OC: Oral cancer
OPC: Oropharyngeal cancer
OR: Odds ratio
PC: Principal component
PheWAS: Phenome-wide association study
PMBB: Penn Medicine Biobank
PRS: Polygenic risk score
QC: Quality control
SD: Standard deviation
SNP: Single nucleotide polymorphism
UKBB: UK Biobank
