# Supplementary material for: Polygenic risk score-based phenome-wide association study of head and neck cancer across two large biobanks
Source: BMC Med. 2024 Mar 14;22:120. doi: 10.1186/s12916-024-03305-2 (PMC10941505; doi:10.1186/s12916-024-03305-2)
Supplement: Supplementary file 1 — Additional file 1: Method S1. Penn Medicine Biobank banner author list and contribution statements. Method S2. Detailed definition of HNSCC. Method S3. Detailed information on the genotype data quality control and imputation procedures. Method S4. Generation of polygenic risk scores. Method S5. Number of missing data for each variable in the UK Biobank. Table S1. Characteristics of participants in the UK Biobank. Table S2. Characteristics of participants in the Penn Medicine Biobank. Table S3. Odds ratio for HNSCC and its subtypes associated with genetic risk in the UK Biobank. Table S4. Odds ratio for HNSCC and its subtypes associated with genetic risk across subgroups by age, sex, and smoking status in the UK Biobank. Table S5. Odds ratio for HNSCC and its subtypes associated with genetic risk in the Penn Medicine Biobank. Table S6. Odds ratio for HNSCC associated with genetic risk across different case–control ratios in the UK Biobank and Penn Medicine Biobank. Table S7. The ancestry-specific odds ratio for HNSCC associated with genetic risk in the Penn Medicine Biobank. Figure S1. Study flowchart. Figure S2. Prevalence plot for significant phenotypes in PheWAS according to genetic risk groups. [file 12916_2024_3305_MOESM1_ESM.pdf]

Additional file 1.

Polygenic risk score-based phenome-wide association study of head and neck cancer across two large biobanks. Young Chan Lee, Sang-Hyuk Jung, Manu Shivakumar, Soojin Cha, Woong-Yang Park, Hong-Hee Won, Young-Gyu Eun, Penn Medicine Biobank, Dokyoon Kim.

Supplementary contents

Method S1. Penn Medicine Biobank banner author list and contribution statements.
Method S2. Detailed definition of HNSCC.
Method S3. Detailed information on the genotype data quality control and imputation procedures.
Method S4. Generation of polygenic risk scores.
Method S5. Number of missing data for each variable in the UK Biobank.

Table S1. Characteristics of participants in the UK Biobank.
Table S2. Characteristics of participants in the Penn Medicine Biobank.
Table S3. Odds ratio for HNSCC and its subtypes associated with genetic risk in the UK Biobank.
Table S4. Odds ratio for HNSCC and its subtypes associated with genetic risk across subgroups by age, sex, and smoking status in the UK Biobank.
Table S5. Odds ratio for HNSCC and its subtypes associated with genetic risk in the Penn Medicine Biobank.
Table S6. Odds ratio for HNSCC associated with genetic risk across different case-control ratios in the UK Biobank and Penn Medicine Biobank.
Table S7. The ancestry-specific odds ratio for HNSCC associated with genetic risk in the Penn Medicine Biobank.
Table S8. Full results of HNSCC PRS-PheWAS in UK Biobank and Penn Medicine Biobank.
Table S9. Full results of OPC PRS-PheWAS in UK Biobank and Penn Medicine Biobank.
Table S10. Full results of OC PRS-PheWAS in UK Biobank and Penn Medicine Biobank.

*__Tables S8-10__ are provided in Additional file 2 (as an Excel file).

Figure S1. Study flowchart.
Figure S2. Prevalence plot for significant phenotypes in PheWAS according to genetic risk groups.

Method S1. Penn Medicine Biobank banner author list and contribution statements.

PMBB Leadership Team

Daniel J. Rader, M.D., Marylyn D. Ritchie, Ph.D.

Contributions: All authors contributed to securing funding, study design, and oversight. All authors reviewed the final version of the manuscript.

Patient Recruitment and Regulatory Oversight

JoEllen Weaver, Nawar Naseer, Ph.D., M.P.H., Giorgio Sirugo, M.D., Ph.D., Afiya Poindexter, Yi-An Ko, Ph.D., Kyle P. Nerz

Contributions: JW manages patient recruitment and regulatory oversight of the study. NN manages participant engagement, assists with regulatory oversight, and researcher access. GS assists with researcher access. AP, YK, KPN perform recruitment and enrollment of study participants.

Lab Operations

JoEllen Weaver, Meghan Livingstone, Fred Vadivieso, Stephanie DerOhannessian, Teo Tran, Julia Stephanowski, Salma Santos, Ned Haubein, Ph.D., Joseph Dunn

Contributions: JW, ML, FV, SD conduct oversight of lab operations. ML, FV, AK, SD, TT, JS, SS perform sample processing. NH, JD are responsible for sample tracking and the laboratory information management system.

Clinical Informatics

Anurag Verma, Ph.D., Colleen Morse Kripke, M.S. DPT, MSA, Marjorie Risman, M.S., Renae Judy, B.S., Colin Wollack, M.S.

Contributions: All authors contributed to the development and validation of clinical phenotypes used to identify study subjects and (when applicable) controls.

Genome Informatics

Anurag Verma Ph.D., Shefali S. Verma, Ph.D., Scott Damrauer, M.D., Yuki Bradford, M.S., Scott Dudek, M.S., Theodore Drivas, M.D., Ph.D.

Contributions: AV, SSV, and SD are responsible for the analysis, design, and infrastructure needed to quality control genotype and exome data. YB performs the analysis. TD and AV provide variant and gene annotations and their functional interpretation of variants.

68 **Method S2.** Detailed definition of HNSCC.

Study reference	Definition	Detail criteria§		
		HNSCC cases	OPC cases	OC cases
**GAME-ON** (Derivation set for PRS generation)	(1) HNSCC (head and neck squamous cell carcinoma)<br>(2) OPC (oropharynx)<br>(3) OC (oral cavity)	**ICD-10 codes:**<br>Union of Oropharynx, Oral cavity, and hypopharynx (C13.0–C13.9) and overlapping (C14 and combination of the codes for other sites); 25 oral or pharyngeal cases had unknown ICD codes (others)	**ICD-10 codes:**<br>oropharynx (C01.9, C02.4 and C09.0–C10.9).	**ICD-10 codes:**<br>oral cavity (C02.0–C02.9, C03.0–C03.9, C04.0–C04.9 and C05.0–C06.9).
**UK Biobank** (Validation set in this study)	(1) HNSCC<br>(2) OPC<br>(3) OC	**ICD-9 codes:**<br>Union of Oropharynx, Oral cavity, and larynx (1610-1619)<br><br>**ICD-10 codes:**<br>Union of Oropharynx, Oral cavity, hypopharynx (C12.9, C13.0–C13.2, C13.8, and C13.9) and larynx (C32.0–C32.3, C32.8 and C32.9)	**ICD-9 codes:**<br>Oropharynx (1453, 1460,1461)<br><br>**ICD-10 codes:**<br>Oropharynx (C01, C02.0, C02.4, C05.1, C05.2, C09.0-C10.9, C14.0).	**ICD-9 codes:**<br>Oral cavity (140.0–140.9, 141.0–141.9, 142.0–142.8, 143.0–143.9, 144.0–144.9, 145.0–145.9, and 230.0)<br>**ICD-10 codes:**<br>Oral cavity (C00.0- C00.9, C02.0–C02.9, C03.0–C03.9, C04.0–C04.9, C05.0–C06.9, C148).
**Penn Medicine Biobank** (Replication set in this study)	(1) HNSCC<br>(2) OPC<br>(3) OC	**ICD-9 codes:**<br>Union of Oropharynx and Oral cavity.<br><br>**ICD-10 codes:**<br>Union of Oropharynx and Oral cavity.	**ICD-9 codes:**<br>Oropharynx (146.0–149.9)<br><br>**ICD-10 codes:**<br>Oropharynx (C01.9, C02.4 and C09.0–C10.9).	**ICD-9 codes:**<br>Oral cavity (140.0–140.9, 141.0–141.9, 142.0–142.8, 143.0–143.9, 144.0–144.9, 145.0–145.9, and 230.0)<br><br>**ICD-10 codes:**<br>Oral cavity (C02.0–C02.9, C03.0–C03.9, C04.0–C04.9 and C05.0–C06.9).

69 Abbreviations: GAME-ON, Genetic Associations and Mechanisms in Oncology; ICD,
70 International Statistical Classification of Diseases and Related Health Problems; HNSCC, head
71 and neck squamous cell carcinoma; OC, oral cavity cancer; OPC, oropharynx cancer.

Method S3. Detailed information on the genotype data quality control and imputation procedures.

UK Biobank

UK Biobank samples (version 3; March 2018) were genotyped for > 800,000 SNPs using either the Affymetrix UK BiLEVE Axiom array or the Affymetrix UK Biobank Axiom array. Imputation was carried out centrally by UK Biobank researchers using the merged 1000 Genomes Project panel and UK 10K panel; SHAPEIT3 was used for phasing and IMPUTE2 was used for imputation (GRCh37/hg19) [14, 15]. After imputation, variant-level quality control (QC) was performed by filtering SNPs on two criteria: (i) minor allele frequency < 1%, (ii) imputation quality score (INFO) < 0.3, and (iii) the Hardy–Weinberg equilibrium with a *P*-value of $< 10^{-6}$. A total of 9,505,768 imputed autosomal SNPs passed the QC criteria. Sample-level QC was performed by excluding samples on the basis of (i) participants identified as not of 'White-British' ancestry according to either self-report or principal components (PC) analysis of genetic ancestry, (ii) mismatched sex, and (iii) having second-degree or closer relatives also in the Biobank. After exclusion, 308,492 White-British participants were determined eligible for the genetic analyses.

Penn Medicine Biobank

Penn Medicine Biobank consists of 43,623 samples that have been genotyped by the GSA genotyping array. We performed genotype imputations for two Penn Medicine Biobank datasets using Eagle2 [16] and Minimac4 [17] softwares on TOPMed Imputation Server [18]. Imputation was performed for all autosomes, with TOPMed version R2 on GRCh38 reference panel [19]. After imputation, variant-level QC was performed by filtering SNPs on three criteria: (ii) minor allele frequency <0.01, (ii) marker call rate <0.05, and (iii) INFO <0.2. Sample-level QC was performed by excluding samples on the basis of (i) mismatched sex or (ii) having second-degree or closer relatives also in the Biobank. We inferred ancestry by projecting array genotype data onto PC axes defined by individuals from the HapMap3 [20]. Then, we performed a kernel density estimator (KDE) algorithm on all samples to determine their genetically informed ancestry. We trained a KDE using the HapMap3 PCs and used the KDEs to calculate the likelihood of a given sample belonging to each of the five continental ancestry groups. Samples were excluded from analysis if no ancestry likelihoods were greater than 0.3, or if more than three ancestry likelihoods were greater than 0.3. After exclusion, a total of 27,933 individuals considered European (non-Hispanic White) ancestry and 10,468 individuals considered African American (non-Hispanic Black) ancestry were determined eligible for the replication analyses.

Method S4. Generation of polygenic risk scores.

To generate polygenic risk scores (PRSs), we utilized the genome-wide association study (GWAS) summary statistics from the GAME-ON (https://www.ncbi.nlm.nih.gov/projects/gap/cgi-bin/study.cgi?study_id=phs001202.v1.p1) Network. The HNSCC, OPC, and OC cases were identified based on the following ICD-10 codes: oral cavity (C02.0–C02.9, C03.0–C03.9, C04.0–C04.9, and C05.0–C06.9) and oropharynx (C01.9, C02.4, and C09.0–C10.9). The GWASs (HNSCC [5,974 cases and 4,012 controls], OPC [2,617 cases and 4,012 controls], and OC [2,958 cases and 4,012 controls]). The GWASs were performed using PLINK 1.90 with sex, age, 10 PCs, and genotyping batch as covariates. The genotype data for the oral and pharyngeal OncoArray study can be downloaded from the database of Genotypes and Phenotypes (dbGaP) under accession phs001202.v1.p1. Of note, the GWASs did not include the additional external controls (2,476 shared controls [1,453 from the EPIC study and 1,023 from the Toronto study]) beyond the GAME-ON data used by Lesseur, Corina, et al. [21] in their GWAS analysis.

We constructed PRSs for HNSCC, OPC, and OC by using a Bayesian polygenic prediction method, PRS-CS [22], which infers the posterior mean effect size of each variant using the linkage disequilibrium (LD) reference panel and GWAS summary. The 1000G Project phase 3 EUR data was used to be the external LD reference panel. The posterior SNP effect sizes in PRS-CS were inferred from GAME-ON summary statistics, with default settings, and automatic estimation of the global shrinkage parameter (PRS-CS-auto). The individual PRSs were computed from beta coefficients as the weighted sum of the risk alleles by applying PLINK version 1.90 with the –score command [23]. The detailed number of SNPs used in the analysis is depicted as follows (**Table**).

Table. Number of used SNPs in generating PRSs.

	GAME-ON (GWAS)	1000G hapmap3 (as reference SNPs)	UK Biobank	Penn Medicine Biobank
**PRS**	**Base set**	**LD reference panel**	**Discovery set**	**Replication set**
HNSCC PRS	9,396,590	1,120,697	972,182	967,888
OPC PRS	9,396,590	1,120,697	972,182	967,888
OC PRS	9,396,590	1,120,697	972,182	967,888

Abbreviations: GWAS, genome-wide association study; SNP, single nucleotide polymorphism; PRS, polygenic risk score; HNSCC, head and neck squamous cell carcinoma; OC, oral cavity cancer; OPC, oropharynx cancer; LD, linkage disequilibrium.

129 **Method S5.** Number of missing data for each variable in the UK Biobank.

**Variable**	**Field ID**	**No. of missing data (%)**
**Total number of participants**	n/a	**(n=308,492)**
**Smoking**		
Current tobacco smoking	1239	206 (0.07%)
Past tobacco smoking	1249	24,417 (7.91%)
Maternal smoking around birth	1787	43,425 (14.08%)
Number of cigarettes previously smoked daily	2887	231,892 (75.17%)
Age stopped smoking	2897	227,870 (73.87%)
Number of unsuccessful stop-smoking attempts	2926	234,527 (76.02%)
Smoking status	20116	1,143 (0.37%)
Pack years of smoking	20161	209,584 (67.94%)
**Alcohol**		
Alcohol intake frequency	1558	0 (0.00%)
Alcohol usually taken with meals	1618	143,325 (46.46%)
Alcohol intake versus 10 years previously	1628	23,247 (7.54%)
Alcohol drinker status	20117	332 (0.11%)
Amount of alcohol drunk on a typical drinking day	20403	221,464 (71.79%)
Ever physically dependent on alcohol	20404	306,335 (99.30%)
Ever had known person concerned about, or recommend reduction of, alcohol consumption	20405	213,001 (69.05%)
Frequency of consuming six or more units of alcohol	20416	221,259 (71.72%)
Other non-alcoholic drinks	100510	180,608 (58.55%)
**HPV**		
HPV type-16	23075	302,570 (98.08%)

130 Abbreviation: HPV, Human papillomavirus.

Table S1. Characteristics of participants in the UK Biobank.

	**Total (n=308,492)**	**Control (n= 306,739)**	**HNSCC case (n=1,753)**	***P*-value***
**Age, mean ± SD**	58.0 ± 7.9	58.0 ± 7.9	59.9 ± 6.9	<.001
**Sex, No. (%)**				<.001
**Male**	140,232 (45.5%)	139,052 (45.3%)	1,180 (67.3%)	
**Female**	168,260 (54.5%)	167,687 (54.7%)	573 (32.7%)	
**HNSCC subtypes, No. (%)**				n/a
**OPC**	556 (0.2%)	n/a	556 (31.7%)	
**OC**	856 (0.3%)	n/a	856 (48.8%)	
**Others**	341 (0.1%)	n/a	341 (19.5%)	
**Smoking status, No. (%)**				<.001
Never	162,723 (52.9%)	162,142 (53.1%)	581 (33.4%)	
Previous	112,322 (36.5%)	111,527 (36.5%)	795 (45.7%)	
Current	32,304 (10.5%)	31,939 (10.5%)	365 (21.0%)	
**Alcohol intake frequency, No. (%)**				<.001
Daily or almost daily	23,107 (7.5%)	22,943 (7.5%)	164 (9.4%)	
Three or four times a week	60,402 (19.6%)	59,925 (19.5%)	477 (27.2%)	
Once or twice a week	71,160 (23.1%)	70,788 (23.1%)	372 (21.2%)	
One to three times a month	82,213 (26.7%)	81,776 (26.7%)	437 (24.9%)	
Special occasions only	35,678 (11.6%)	35,537 (11.6%)	141 (8.0%)	
Never	35,887 (11.6%)	35,725 (11.6%)	162 (9.2%)	
**HPV positivity, No. (%)**				<.001
**Positive**	276 (4.7%)	267 (4.5%)	9 (26.5%)	
**Negative**	5,646 (95.3%)	5,621 (95.5%)	25 (73.5%)	

*****P*-value indicates the significance of the difference between the control and HNSCC case groups. Abbreviations: HPV, Human papillomavirus; HNSCC, head and neck squamous cell carcinoma; OC, oral cavity cancer; OPC, oropharynx cancer; SD, standard deviation.

Table S2. Characteristics of participants in the Penn Medicine Biobank.

	**Total (n=38,401)**	**Control (n=37,670)**	**HNSCC case (n=731)**	***P*-value***
**Age, mean ± SD**	55.9 ± 16.4	55.7 ± 16.4	63.4 ± 11.0	<.001
**Sex, No. (%)**				<.001
**Male**	19,165 (49.9%)	18,614 (49.4%)	551 (75.4%)	
**Female**	19,236 (50.1%)	19,056 (50.6%)	180 (24.6%)	
**Ancestry, No. (%)**				<.001
**European**	27,933 (72.7%)	27,276 (72.4%)	657 (89.9%)	
**African American**	10,468 (27.3%)	10,394 (27.6%)	74 (10.1%)	
**HNSCC subtypes, No. (%)**				n/a
**OPC**	231 (0.6%)	n/a	231 (31.6%)	
**OC**	437 (1.1%)	n/a	437 (59.8%)	
**Others**	64 (0.2%)	n/a	64 (8.8%)	

*****_P_-value indicates the significance of the difference between the control and HNSCC case groups. Abbreviations: HNSCC, head and neck squamous cell carcinoma; OC, oral cavity cancer; OPC, oropharynx cancer; SD, standard deviation.

Table S3. Odds ratio for HNSCC and its subtypes associated with genetic risk in the UK Biobank.

			HNSCC PRS			OPC PRS			OC PRS		
**Outcome**	**Total no. of participants**	**No. of cases (%)**	**OR perSD increase (95% CI)**	***P*-value**	***Variance explained, %**	**OR perSD increase (95% CI)**	***P*-value**	***Variance explained, %**	**OR perSD increase (95% CI)**	***P*-value**	***Variance explained, %**
**HNSCC**	308,492	1,753 (0.57%)	1.12 (1.06-1.17)	<.001	0.38	1.10 (1.05-1.16)	<.001	0.36	1.09 (1.04-1.15)	<.001	0.34
**OPC**	307,295	556 (0.18%)	1.18 (1.08-1.28)	<.001	0.47	1.20 (1.10-1.31)	<.001	0.51	1.10 (1.01-1.20)	.027	0.35
**OC**	307,295	856 (0.28%)	1.10 (1.02-1.17)	.009	0.27	1.07 (1.00-1.15)	.058	0.24	1.09 (1.02-1.17)	.015	0.26

All analyses were adjusted by age, sex, genotype array, and PC 1 to 10.

*The proportion of variance explained for PRS alone was computed as Nagelkerke's pseudo-R2.

Abbreviations: HNSCC, head and neck squamous cell carcinoma; OC, oral cavity cancer; OPC, oropharynx cancer; PRS, polygenic risk score; SD, standard deviation; OR, Odds ratio; CI, confidence interval; PC, principal component.

Table S4. Odds ratio for HNSCC and its subtypes associated with genetic risk across subgroups by age, sex, and smoking status in the UK Biobank.

			HNSCC PRS		OPC PRS		OC PRS	
**Subgroup**	**Outcome**	**Total no. of participants/ no. of cases (%)**	**OR perSD increase (95% CI)**	***P*-value**	**OR perSD increase (95% CI)**	***P*-value**	**OR perSD increase (95% CI)**	***P*-value**
**Younger**	HNSCC	166,624/791	1.07 (1.00-1.15)	.059	**1.11 (1.03-1.19)**	**.006**	1.03 (0.95-1.10)	.496
(Age ≤ 60 years)	OPC	166,624/323	**1.13 (1.01-1.27)**	**.032**	**1.24 (1.11-1.38)**	**<.001**	1.01 (0.90-1.13)	.865
	OC	166,624/396	1.05 (0.95-1.16)	.375	1.05 (0.95-1.16)	.323	1.03 (0.94-1.14)	.507
**Elderly**	HNSCC	141,868/962	**1.16 (1.08-1.23)**	**<.001**	**1.10 (1.03-1.17)**	**.004**	**1.15 (1.08-1.23)**	**<.001**
(Age > 60 years)	OPC	141,868/233	**1.25 (1.09-1.43)**	**<.001**	**1.16 (1.01-1.32)**	**.029**	**1.24 (1.09-1.41)**	**.001**
	OC	141,868/460	**1.14 (1.04-1.25)**	**.006**	1.08 (0.99-1.19)	.091	**1.14 (1.04-1.25)**	**.007**
**Male**	HNSCC	140,232/1,180	**1.12 (1.06-1.19)**	**<.001**	**1.10 (1.04-1.17)**	**.002**	**1.08 (1.02-1.15)**	**.008**
	OPC	140,232/404	**1.18 (1.06-1.30)**	**.001**	**1.23 (1.11-1.36)**	**<.001**	1.08 (0.98-1.19)	.131
	OC	140,232/513	1.08 (0.99-1.18)	.079	1.07 (0.98-1.17)	.130	1.06 (0.97-1.16)	.182
**Female**	HNSCC	168,260/573	**1.11 (1.02-1.21)**	**.014**	**1.11 (1.02-1.20)**	**.017**	**1.11 (1.02-1.21)**	**.013**
	OPC	168,260/152	**1.18 (1.00-1.24)**	**.045**	1.13 (0.96-1.32)	.156	1.16 (0.98-1.36)	.077
	OC	168,260/343	**1.12 (1.00-1.24)**	**.048**	1.07 (0.96-1.19)	.251	**1.13 (1.01-1.26)**	**.028**
**Never-smoker**	HNSCC	119,038/413	**1.10 (1.00-1.22)**	**.050**	1.08 (0.98-1.19)	.131	**1.11 (1.01-1.23)**	**.036**
	OPC	119,038/119	1.16 (0.96-1.39)	.118	1.16 (0.97-1.40)	.106	1.16 (0.96-1.39)	.123
	OC	119,038/223	1.11 (0.97-1.27)	.128	1.07 (0.94-1.23)	.320	1.13 (0.99-1.30)	.069
**Ever-smoker**	HNSCC	190,562/1,328	**1.12 (1.06-1.18)**	**<.001**	**1.11 (1.05-1.17)**	**<.001**	**1.09 (1.03-1.15)**	**.003**
	OPC	190,562/433	**1.17 (1.06-1.29)**	**.001**	**1.21 (1.10-1.33)**	**<.001**	1.08 (0.98-1.19)	.113
	OC	190,562/628	**1.09 (1.00-1.18)**	**.038**	1.07 (0.98-1.16)	.112	1.07 (0.99-1.16)	.077

All analyses were adjusted by age, sex, genotype array, and PC 1 to 10.

Abbreviations: HNSCC, head and neck squamous cell carcinoma; OC, oral cavity cancer; OPC, oropharynx cancer; PRS, polygenic risk score; SD, standard deviation; OR, Odds ratio; CI, confidence interval; PC, principal component.

Table S5. Odds ratio for HNSCC and its subtypes associated with genetic risk in the Penn Medicine Biobank.

			**HNSCC PRS**			**OPC PRS**			**OC PRS**		
**Outcome**	**Total no. of participants**	**No. of cases (%)**	**OR perSD increase (95% CI)**	***P*-value**	***Variance explained, %**	**OR perSD increase (95% CI)**	***P*-value**	***Variance explained, %**	**OR perSD increase (95% CI)**	***P*-value**	***Variance explained, %**
**HNSCC**	38,401	731 (1.90%)	1.17 (1.07-1.26)	<.001	2.46	1.25 (1.09-1.44)	.002	2.42	1.12 (1.01-1.25)	.027	2.42
**OPC**	37,901	231 (0.61%)	1.13 (1.05-1.22)	.002	2.60	1.24 (1.08-1.42)	.002	2.63	1.11 (1.00-1.22)	.040	2.46
**OC**	38,107	437 (1.15%)	1.14 (1.06-1.23)	<.001	2.56	1.18 (1.03-1.35)	.017	2.56	1.08 (0.98 -1.19)	.136	2.51

All analyses were adjusted by age, sex, ethnicity, and PC 1 to 10.

*The proportion of variance explained for PRS alone was computed as Nagelkerke's pseudo-R2.

Abbreviations: HNSCC, head and neck squamous cell carcinoma; OC, oral cavity cancer; OPC, oropharynx cancer; PRS, polygenic risk score; SD, standard deviation; OR, Odds ratio; CI, confidence interval; PC, principal component.

Table S6. Odds ratio for HNSCC associated with genetic risk across different case-control ratios in the UK Biobank and Penn Medicine Biobank.

	UK Biobank[1]				Penn Medicine Biobank[2]			
**Ratio[*] (case:control)**	**Total no. of controls**	**OR perSD increase (95% CI)**	***P*-value**	**[**]Variance explained, %**	**Total no. of controls**	**OR perSD increase (95% CI)**	***P*-value**	**[**]Variance explained, %**
**full**	306,739	1.12 (1.06-1.17)	<.001	0.38	37,670	1.17 (1.07-1.26)	<.001	2.46
**1:10**	17,530	1.10 (1.05-1.16)	<.001	0.88	7,310	1.14 (1.05-1.24)	.002	2.57
**1:5**	8,765	1.08 (1.03-1.14)	.003	1.10	3,655	1.16 (1.06-1.26)	.001	3.47
**1:3**	5,259	1.09 (1.03-1.16)	.002	1.28	2,193	1.16 (1.06-1.27)	.002	4.16
**1:1**	1,753	1.09 (1.02-1.17)	.013	1.72	731	1.15 (1.02-1.29)	.012	4.85

[1] The UK Biobank analyses were adjusted by age, sex, genotype array, and PC 1 to 10.

[2] The Penn Medicine Biobank analyses were adjusted by age, sex, ethnicity, and PC 1 to 10.

[*]Controls were extracted from samples matched for age and sex with cases for each ratio using the "matchIt" R package.

[**]The proportion of variance explained for PRS alone was computed as Nagelkerke's pseudo-R2.

Abbreviations: HNSCC, head and neck squamous cell carcinoma; OC, oral cavity cancer; OPC, oropharynx cancer; PRS, polygenic risk score; SD, standard deviation; OR, Odds ratio; CI, confidence interval; PC, principal component.

Table S7. The ancestry-specific odds ratio for HNSCC associated with genetic risk in the Penn Medicine Biobank.

Cohort	Ancestry	Total no. of participants	No. of cases (%)	OR perSD increase (95% CI)	*P*-value	*Variance explained, %
**PMBB**	European	27,933	657 (2.35%)	1.14 (1.05-1.24)	.001	4.44
	African American	10,468	74 (0.71%)	1.27 (1.01-1.60)	.045	6.66

All analyses were adjusted by age, sex, and PC 1 to 10.

*The proportion of variance explained for PRS alone was computed as Nagelkerke's pseudo-R2.

Abbreviations: PMBB, Penn Medicine Biobank; HNSCC, head and neck squamous cell carcinoma; SD, standard deviation; OR, Odds ratio; CI, confidence interval; PC, principal component.

Table S8. Full results of HNSCC PRS-PheWAS in UK Biobank and Penn Medicine Biobank.

Table S9. Full results of OPC PRS-PheWAS in UK Biobank and Penn Medicine Biobank.

Table S10. Full results of OC PRS-PheWAS in UK Biobank and Penn Medicine Biobank.

*__Tables S8-10__ are provided in Additional file 2 (as an Excel file).

Figure S1. Study flowchart.

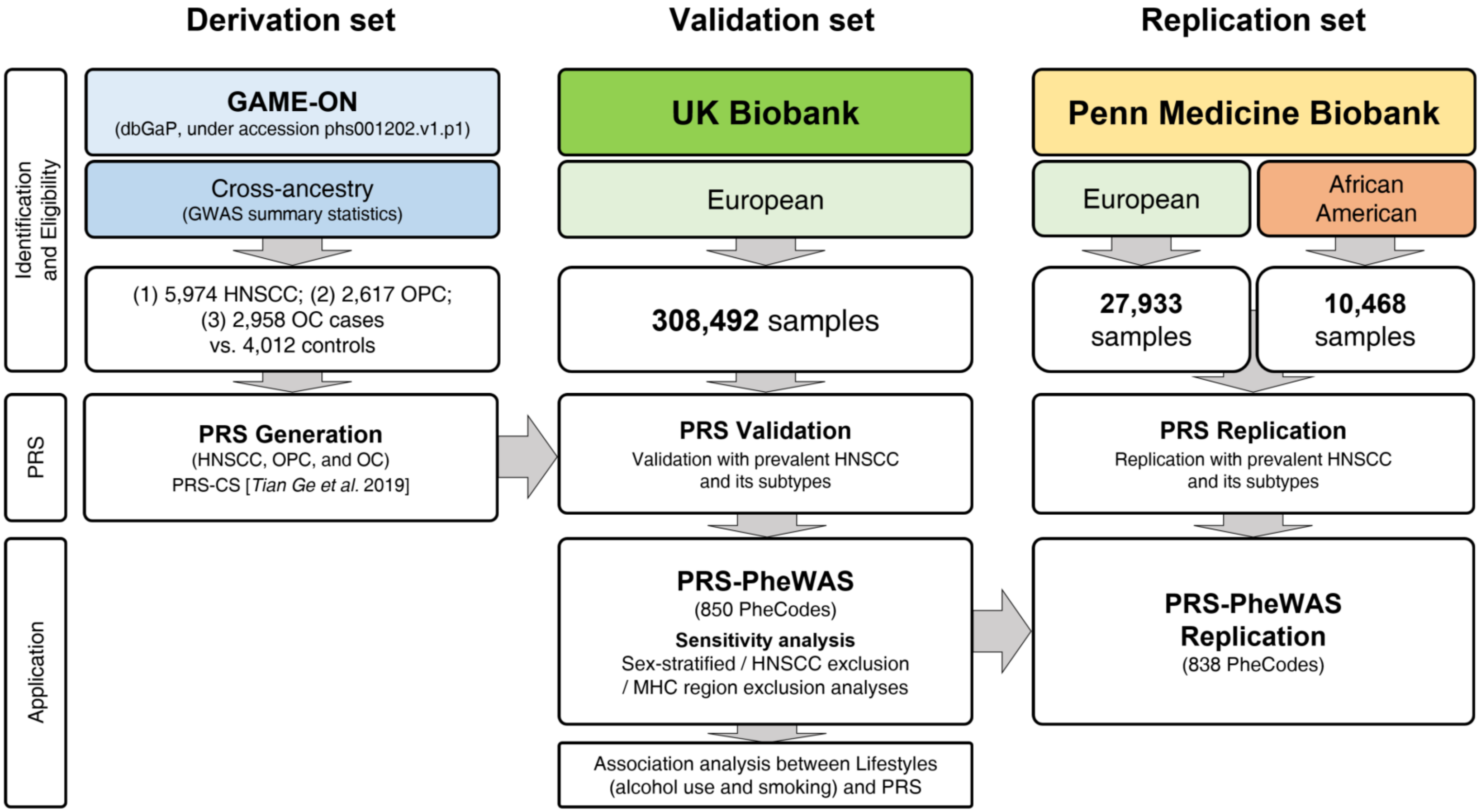

Abbreviations: HNSCC, head and neck squamous cell carcinoma; OC, oral cavity cancer; OPC, oropharynx cancer; PRS, polygenic risk score.

Figure S2. Prevalence plot for significant phenotypes in PheWAS according to genetic risk groups.

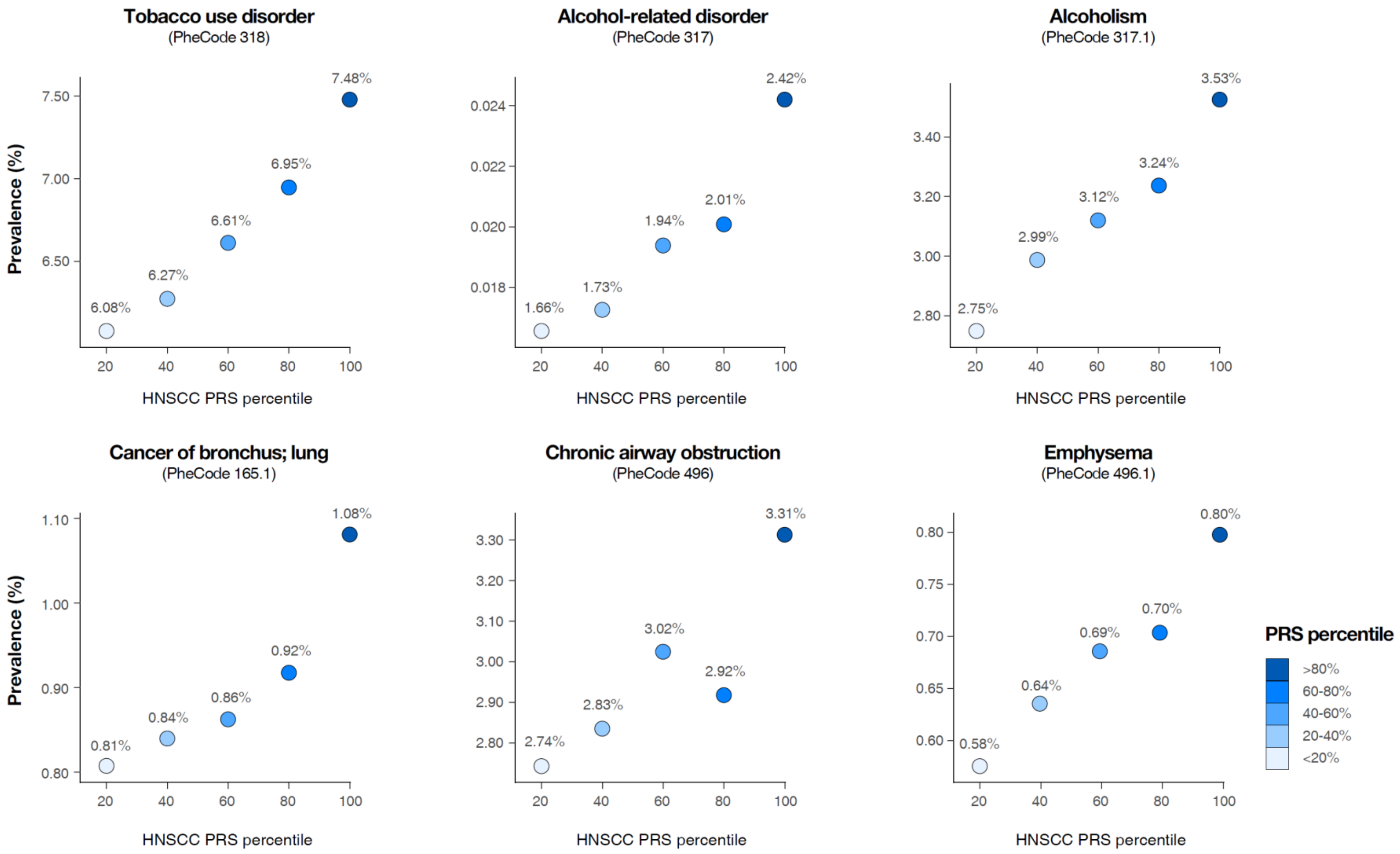

Abbreviations: HNSCC, head and neck squamous cell carcinoma; PRS, polygenic risk score.
